# Inhibition of autophagy exerts anti-colon cancer effects via apoptosis induced by p53 activation and ER stress

**DOI:** 10.1186/s12885-015-1789-5

**Published:** 2015-10-24

**Authors:** Kosuke Sakitani, Yoshihiro Hirata, Yohko Hikiba, Yoku Hayakawa, Sozaburo Ihara, Hirobumi Suzuki, Nobumi Suzuki, Takako Serizawa, Hiroto Kinoshita, Kei Sakamoto, Hayato Nakagawa, Keisuke Tateishi, Shin Maeda, Tsuneo Ikenoue, Shoji Kawazu, Kazuhiko Koike

**Affiliations:** 1 The Institute for Adult Diseases, Asahi Life Foundation, 2-2-6 Bakuro-cho, Nihon-Bashi, Chuo-ku Tokyo, 113-8655 Japan; 2 Department of Gastroenterology, Graduate School of Medicine, The University of Tokyo, Tokyo, Japan; 3 Gastroenterology Division, Yokohama City University Graduate School of Medicine, Yokohama, Japan; 4 Division of Clinical Genome Research, The Institute of Medical Science, The University of Tokyo, Tokyo, Japan

**Keywords:** Autophagy, Colon cancer, Apoptosis

## Abstract

**Background:**

Although some molecularly targeted drugs for colorectal cancer are used clinically and contribute to a better prognosis, the current median survival of advanced colorectal cancer patients is not sufficient. Autophagy, a basic cell survival mechanism mediated by recycling of cellular amino acids, plays an important role in cancer. Recently, autophagy has been highlighted as a promising new molecular target. The unfolded protein response (UPR) reportedly act in complementary fashion with autophagy in intestinal homeostasis. However, the roles of UPR in colon cancer under autophagic inhibition remain to be elucidated. We aim to clarify the inhibitory effect of autophagy on colon cancer.

**Methods:**

We crossed *K19*^*CreERT*^ and *Atg5*^*flox/flox*^ mice to generate *Atg5*^*flox/flox*^*/K19*^*CreERT*^ mice. *Atg5*^*flox/flox*^*/K19*^*CreERT*^ mice were first treated with azoxymethane/dextran sodium sulfate and then injected with tamoxifen to inhibit autophagy in CK19-positive epithelial cells. To examine the anti-cancer mechanisms of autophagic inhibition, we used colon cancer cell lines harboring different p53 gene statuses, as well as small interfering RNAs (siRNAs) targeting Atg5 and immunoglobulin heavy-chain binding protein (BiP), a chaperone to aid folding of unfolded proteins.

**Results:**

Colon tumors in *Atg5*^*flox/flox*^*/K19*^*CreERT*^ mice showed loss of autophagic activity and decreased tumor size (the total tumor diameter was 28.1 mm in the control and 20.7 mm in *Atg5*^*flox/flox*^*/K19*^*CreERT*^ mice, *p* = 0.036). We found that p53 and UPR/endoplasmic reticulum (ER) stress-related proteins, such as cleaved caspase 3, and CAAT/enhancer-binding protein homologous protein, are up-regulated in colon tumors of *Atg5*^*flox/flox*^*/K19*^*CreERT*^ mice. Although Atg5 and BiP silencing, respectively, increased apoptosis in p53 wild type cells, Atg5 silencing alone did not show the same effect on apoptosis in p53 mutant cells. However, co-transfection of Atg5 and BiP siRNAs led to increased apoptosis in p53 mutant cells.

**Conclusions:**

Blocking autophagy has potential in the treatment of colon cancer by inducing apoptosis via p53 and ER stress, and suppressing the UPR pathway is a valid strategy to overcome resistance to autophagic inhibition.

## Background

Colorectal cancer is the second leading cause of cancer-related death in the United States [1]. Although some molecularly targeted drugs, such as anti-vascular endothelial cell growth factor (VEGF) and anti-epidermal growth factor receptor (EGFR) antibodies, are used clinically and contribute to a better prognosis, the current median survival of stage IV colorectal cancer patients receiving chemotherapy is shorter than 3 years [2]. Macroautophagy (hereafter referred to as autophagy), which is a highly conserved cell survival mechanism mediated by the recycling of cellular amino acids [3], has been highlighted as a promising new molecular target [3, 4]. For example, chloroquine (CQ), originally developed as an anti-malarial agent, prevents autophagic activity and has been used in several clinical trials of cancer treatment as an autophagic inhibitor [5, 6].

Autophagy is typically activated under conditions of amino acid starvation, and autophagy marker proteins include light chain 3 (LC3) and p62 [5]. p62 (also called SQSTM1) is a selective substrate of autophagy, and its accumulation is observed when autophagy is inhibited [7]. This p62 dysregulation is reportedly associated with proliferation of cancer, including colorectal cancer [8, 9]. Preclinical evidence has shown that the role of autophagy in cancer differs depending on the situation. For instance, although autophagic inhibition promotes cancer initiation, it can suppress growth of some malignancies, such as lung cancer and hepatocellular carcinoma [10–12]. Quite recently, Rosenfeldt et al. showed that the effect of autophagic inhibition on pancreatic cancer depends on p53 status [13].

The endoplasmic reticulum (ER) is an organelle involved in protein folding, and ER stress refers to the condition leading to accumulation of misfolded proteins. The unfolded protein response (UPR), which is the cellular response to increased ER stress, is a basic mechanism of maintaining cell homeostasis [14, 15]. Under ER stress, immunoglobulin heavy-chain binding protein (BiP) and phosphorylated eukaryotic initiation factor 2α (eIF2α) are upregulated [16, 17]. BiP acts as a chaperone to aid folding of unfolded proteins, and eIF2α suppresses general mRNA translation. Severe ER stress can induce apoptotic cell death, and CAAT/enhancer-binding protein homologous protein (CHOP) and c-Jun N-terminal kinase (JNK) have been reported to play a critical role in the induction of apoptosis [18]. Recently, Adolph et al. showed that autophagy and UPR act in complementary fashion in Paneth cells to maintain intestinal homeostasis [19]. However, the roles of autophagy and UPR in colon cancer remain to be elucidated.

To investigate the involvement of autophagy in colon cancer *in vivo*, we treated mice mutant for *Atg5*, an indispensable gene for autophagy [20], with azoxymethane/dextran sodium sulfate (AOM/DSS), which is an established animal model used to induce and analyze colon cancer [21, 22]. Since systemic Atg5 deletion causes neonatal lethality [23], we used *K19*^*CreERT*^ mice to inhibit autophagy specifically in CK19 positive-cell which is known as a marker of epithelial cell [24]. In this report, by genetic inhibition of autophagy and CQ treatment, we showed that suppression of autophagy has an anti-colorectal cancer effect via apoptosis induced by p53 activation and ER stress *in vivo* and *in vitro*.

## Methods

### Mice

*K19*^*CreERT*^ mice were kindly provided by Guoqiang Gu (Vanderbilt University, Nashville, TN, USA) [24]. *ROSA26-lox-stop-lox-YFP* reporter (*ROSA-YFP*) mice, obtained from the Jackson Laboratory, were crossed with *K19*^*CreERT*^ mice to generate *K19*^*CreERT*^/*ROSA-YFP* mice. *Atg5*^*flox/flox*^ mice have been described previously [25] and were kindly provided by Dr. Noboru Mizushima (Tokyo University, Tokyo, Japan). *Atg5*^*flox/flox*^ mice were crossed with *K19*^*CreERT*^ mice to generate *Atg5*^*flox/flox*^*/K19*^*CreERT+*^ mice. C57BL/6 J (B6) mice were from CLEA Japan (Tokyo, Japan). All mice used were of the B6 background. For tamoxifen (TAM) treatment, mice were injected with 10 mg/kg TAM (Cayman Chemical, Ann Arbor, MI, USA) intraperitoneally (i.p.) three times (on days 1, 3, and 5). For CQ treatment, mice were injected with 50 mg/kg CQ (Sigma-Aldrich, St. Louis, MO, USA) i.p. at the times indicated. All animal studies were approved by the Animal Care and Use Ethics Committee at the Institute for Adult Diseases, Asahi Life Foundation.

### Tumor induction

*Atg5*^*flox/flox*^*/K19*^*CreERT+*^ (*Atg5-*deficient mice) and *Atg5*^*flox/flox*^ mice (Cre-negative littermates, used as control mice) were injected i.p. with 12.5 mg/kg AOM (Sigma-Aldrich) on day 1. After 5 days, mice received water supplemented with 2.5 % DSS (MP Biomedicals, Irvine, CA, USA) for 5 days, after which the mice were maintained on regular water for 14 days and subjected to two further DSS treatment cycles. On days 60, 62, and 64, the mice were injected i.p. with 10 mg/kg TAM. On day 67, the mice were sacrificed to analyze colon tumors. Macroscopic colon tumors were counted, and the longest diameter of each tumor was measured using a caliper in a blinded fashion.

### Cell lines

Four established colon cancer cell lines, HCT116, SW48, DLD1, and SW837, were used [26, 27]. HCT116 and SW48 cells harbor the wild type p53 gene, while DLD1 and SW837 cells are mutated in the p53 gene [26, 27]. HCT116 cells were maintained in McCoy’s 5A medium containing 10 % fetal bovine serum (FBS). SW48 and SW837 cells were maintained in Leibovitz’s L-15 medium containing 10 % FBS. DLD1 cells were maintained in RPMI 1640 medium containing 10 % FBS. Hank’s Buffered Salt Solution (HBSS) was used to induce amino acid starvation conditions. The cell lines were obtained from the American Type Culture Collection (Baltimore, MD, USA), and all media formulations were obtained from Sigma-Aldrich.

### Antibodies and reagents

The following primary antibodies were used for immunoblotting and immunohistochemistry: anti-Atg5, anti-Atg7, anti-LC3, anti-p62, anti-PARP, anti-cleaved caspase 3, anti-BiP, anti-p53, anti-phospho-eIF2α, anti-phospho-JNK, anti-phospho-Chk1, anti-phospho-p53, anti-actin (all from Cell Signaling, Beverly, MA, USA), anti-CK19, anti-proliferating cell nuclear antigen (PCNA) (both from Santa Cruz Biotechnology, Santa Cruz, CA, USA), anti-Ki67 (Dako, Carpinteria, CA, USA), anti-p53 (Vector Laboratories, Birmingham, CA, USA), anti-CHOP (Thermo Fisher Scientific, Waltham, MA, USA), and anti-yellow fluorescent protein (YFP) (MBL, Tokyo, Japan). CQ diphosphate salt (Sigma-Aldrich) was dissolved in PBS at the indicated concentrations.

### RNA interference

Small interfering RNAs (siRNAs) targeting Atg5 (MISSION siRNA, Sigma-Aldrich) and BiP (Dharmacon siGENOME SMART pool siRNA, GE Healthcare, Pittsburg, PA, USA) or the non-silencing control (5’-AATTCTCCGAACGTGTCACGT-3’) were transfected into cells using Lipofectamine RNAimax (Invitrogen, Waltham, MA, USA) for 72 h. Immunoblotting was used to verify that the siRNAs reduced cellular protein expression by more than 80 %.

### Immunoblotting

Cells or mouse tissues were disrupted in lysis buffer (20 mM Tris, pH 7.5, 150 mM NaCl, 1 mM EDTA, 1 mM EGTA, 1 % Triton X [Sigma-Aldrich], 2.5 mM sodium pyrophosphate, 1 mM glycerophosphate, 1 mM Na_3_VO_4_, 1 μg/ml leupeptin). The lysates were electrophoresed by SDS-PAGE, transferred to a polyvinylidene difluoride membrane (GE Healthcare), and blocked for 1 h in Tris-buffered saline-Tween 20 with 5 % dry milk. The membrane was incubated overnight at 4 °C with the primary antibody and subsequently washed and incubated with a secondary horseradish peroxidase (HRP)-conjugated antibody. The immunocomplexes were detected using a chemiluminescence detection kit (Luminata Classico Western HRP; Merck Millipore, Darmstadt, Germany). Images were obtained using the LAS 4000 image analyzer (Fujifilm, Tokyo, Japan).

### Immunohistochemistry

Formalin-fixed and paraffin-embedded mouse tissues were cut at a thickness of 3 μm, deparaffinized, and incubated in citrate buffer at 95 °C for 40 min for antigen retrieval. Endogenous peroxidase activity was blocked using 3 % H_2_O_2_. The tissue sections were incubated overnight with rabbit primary antibody, followed by a polyclonal goat anti-rabbit immunoglobulins/biotinylated secondary antibody (DAKO) for 30 min, and then exposed to Streptavidin/HRP (DAKO) for 10 min. The Mouse-on-Mouse Immunodetection kit (Vector Laboratories) was used as the mouse primary antibody for mouse tissues, according to the manufacturer’s instructions. The chromogenic reaction was performed using the Liquid DAB Substrate Chromogen System (Dako). YFP expression in the colons of mice was examined by immunofluorescence staining. The tissues were incubated with anti-YFP antibody followed by Alexa Flour 594-conjugated goat anti-rabbit secondary antibody (Molecular Probes, Eugene, OR, USA) for 30 min, and the nuclei were visualized by DAPI staining (Takara, Tokyo, Japan) for 1 min. The proportion of Ki67-positive cells was determined by counting more than 500 cells in three Ki67-concentrated lesions, and the numbers of cleaved caspase 3-positive cells per field were counted.

### Real-time RT-PCR

Total cellular RNA samples were isolated from mouse colon tissues and from HCT116 cells using NucleoSpin RNA II (Takara). The cDNAs were generated from 1-μg total RNA by reverse transcription using Transcriptor Universal cDNA Master (Roche, Branchburg, NJ, USA). The mRNA expression levels of mouse Atg5, interleukin (IL) 1-β, chemokine (C-X-C motif) ligand 1 (CXCL1), p53 upregulated modulator of apoptosis (PUMA), Noxa, Bax, CHOP, BiP, spliced X-box binding protein 1 (sXBP1) and of human Atg5, Ulk1, Atg7, C-X-C chemokine receptor type 4 (CXCR4), SOX9, CD44, CXCL1, IL8, cellular inhibitor of apoptosis protein 1 (cIAP1), PUMA, Noxa, Bax, CHOP, BiP, and spliced XBP1 were determined by quantitative real-time RT-PCR using the LightCycler 480 instrument II real-time PCR System (Roche). GAPDH mRNA was used as an internal control. The primer sequences used are available on request.

### Flow cytometric analysis of apoptosis

Colon cancer cells (1.0 × 10^4^/ml) were seeded into 24-well plates and 24 h later were treated with siRNAs for 72 h or with CQ for 24 h. Cells were detached by trypsinization and exposed to early and late apoptotic detection reagents (GFP Certified Apoptosis Detection Kit, Enzo life Sciences, Farmingdale, NY, USA) for 15 min. Samples were analyzed by flow cytometry using the FL2 channel for Annexin V detection (early apoptosis) and the FL3 channel for PI detection (late apoptosis) using a fluorescence-activated cell sorter (FACS) (accuriC6, BD Biosciences, Ann Arbor, MI, USA).

### Cell growth assay

The extent of cell growth was assessed using the Cell Counting Kit-8 (CCK-8) from Dojindo Laboratories (Kumamoto, Japan). Cells (1.0 × 10^4^/ml) were seeded into 96-well plates (day 0) and 24 h later were transfected with siRNAs (day 1) for 48 h. CCK-8 solution was added to each well for 2 h. The absorbance at 450 nm was determined using a multi-mode reader (SpectraMax, Molecular Devices, Sunnyvale, CA, USA).

### Statistical analysis

Statistical analysis was performed using Welch’s *t*-test, Mann–Whitney *U*-test, and one-way analysis of variance with Dunnett’s multiple comparison test, where appropriate. Differences were considered statistically significant at p < 0.05.

## Results

### CK19 and Atg5 expression in normal colon mucosa and AOM/DSS-derived colon tumors

**Fig. 1:**
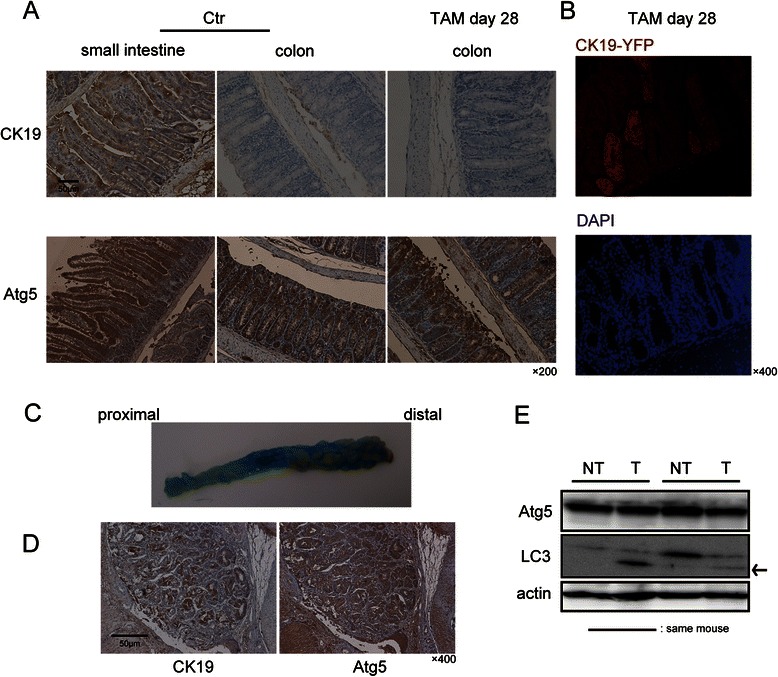
CK19 and Atg5 expression in normal colonic mucosa and azoxymethane/dextran sodium sulphate (AOM/DSS)-derived colon tumors. **a** Representative immunohistochemical images of the small intestine and colon in *Atg5*^*flox/flox*^*/K19*^*CreERT*^ mice before and 28 days after tamoxifen (TAM) injection. Scale bar, 50 μm. Original magnification, ×200. **b** Immunofluorescence analysis of the colon in *K19*^*CreERT*^/*ROSA*-*YFP mice* 28 days after TAM injection; YFP (red) and DAPI (blue) fluorescent staining. Original magnification, ×400. **c** Typical macroscopic examples of colorectal tumors induced by AOM/DSS in *K19*^*CreERT*^ mice before TAM injection. **d** Typical immunohistochemical images of CK19 and Atg5 staining in AOM/DSS-derived colon tumors in *K19*^*CreERT*^ mice before TAM injection. Scale bar, 50 μm. Original magnification, ×400. **e** Immunoblot analysis of Atg5 and LC3 expression in AOM/DSS-derived tumor (T) and non-tumor colon mucosa (NT) in the same *K19*^*CreERT*^ mouse before TAM injection. Actin was used as an internal control. The arrow indicates LC3-II

We first examined the distribution of CK19- and Atg5-positive epithelial cells in normal colonic mucosa of *K19*^*CreERT*^ mice. Even though CK19 exhibited positive staining largely in the small intestinal mucosa, CK19-positive cells were rarely observed, using the conventional immunoperoxidase method, in normal colon mucosa either before or after TAM injection (Fig. 1a, upper panels). To examine the CK19-positive lineage in colon mucosa, we crossed *K19*^*CreERT*^ mice with *ROSA-YFP* reporter mice. In these *K19*^*CreERT*^/*ROSA-YFP* mice, YFP expression is supposed to be induced by Cre-mediated recombination [24]. After TAM injection, approximately 10 % of the colonic mucosa exhibited positive immunohistochemical staining for CK19-YFP (Fig. 1b). In contrast, Atg5 exhibited positive staining in almost the entire cytoplasm of small intestinal and colonic epithelial cells of *Atg5*^*flox/flox*^*/K19*^*CreERT*^ mice before TAM injection. After TAM injection (day 28), approximately 10 % of the normal colonic mucosa was negative for Atg5 (Fig. 1a, lower panels).

Next, we examined the distribution of CK19 and Atg5 in AOM/DSS-derived colon tumors. Macroscopically, as shown in Fig. 1c, approximately 10 colon tumors were present mainly in the middle or distal colon, in line with a previous report [21]. CK19 exhibited global positive staining in the cytoplasm of colorectal tumor cells, as typically observed in human colon cancer [28] (Fig. 1d, left panel). Atg5 also exhibited global positive staining in the colorectal tumor cell cytoplasm (Fig. 1d, right panel). Western blotting showed that LC3-II protein levels were elevated in colon tumor tissues compared with non-tumor colon mucosa (Fig. 1e). Taken together, the proportion of CK19-positive cells and autophagic activity were higher in colon tumors than in normal colon mucosa.

### Autophagic inhibition in AOM/DSS-derived mouse colon tumors using the CreERT system

**Fig. 2:**
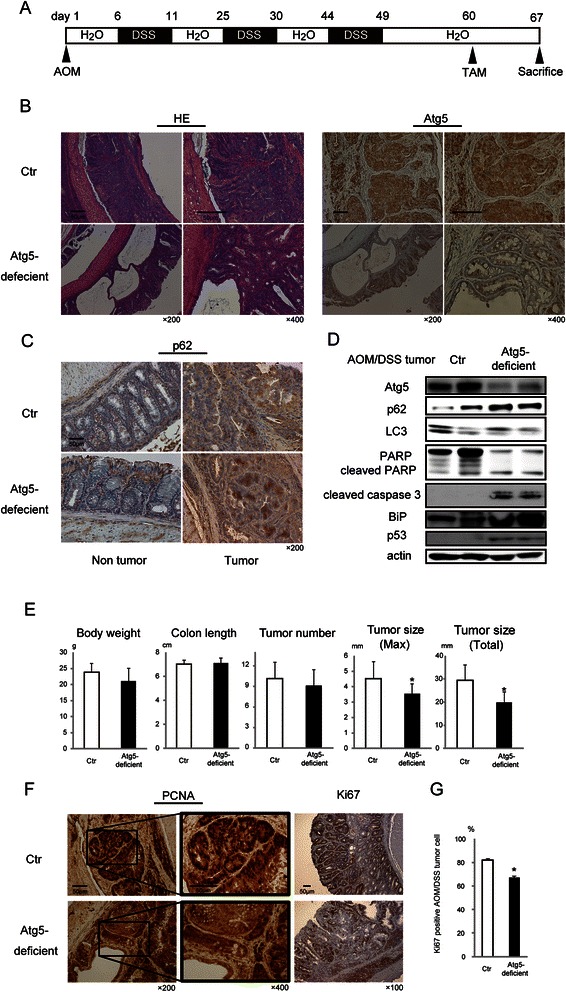
Autophagic inhibition in azoxymethane/dextran sodium sulfate (AOM/DSS)-derived mice colon tumors using the CreERT system. **a** Schematic representation of Atg5 genetic inhibition by tamoxifen (TAM) in AOM/DSS-induced colon tumors. **b** Representative histopathological images of staining for hematoxylin and eosin (HE) or Atg5 in AOM/DSS-derived colon tumors in *Atg5*^*flox/flox*^*/K19*^*CreERT+*^ (*Atg5-*deficient) and *Atg5*^*flox/flox*^ (Ctr) mice 7 days after TAM injection. Scale bars, 50 μm. Original magnification, ×200/400. **c** Representative immunohistochemical images of p62 staining in the non-tumor colon mucosa and AOM/DSS-derived colon tumors in *Atg5-*deficient mice and Ctr mice. Scale bar, 50 μm. Original magnification, ×200. **d** Representative immunoblot analysis image of the indicated proteins from AOM/DSS-derived colon tumors in *Atg5-*deficient mice and Ctr mice (two different mice each). **e** Body weight, colon length, tumor number, and tumor size were determined in Ctr mice (*n* = 9) and *Atg5-*deficient mice (*n* = 11). Data shown are means and SEM. *; *p* < 0.05 by the Mann–Whitney *U*-test. **f** Representative immunohistochemical images for PCNA and Ki67 staining in AOM/DSS-derived colon tumors in Ctr mice and *Atg5-*deficient mice. Scale bars, 50 μm. Original magnification, ×100/200/400. **g** The proportion of Ki67-positive cells in AOM/DSS-derived tumor cells in Ctr mice and *Atg5-*deficient mice. Data shown are means ± SEM (*n* = 3). *; *p* < 0.05 by the Mann–Whitney *U*-test

Next, we suppressed Atg5 gene expression in established colon tumors. As shown in the schematic chart in Fig. 2a, *Atg5*^*flox/flox*^*/K19*^*CreERT+*^ (*Atg5-*deficient mice) and *Atg5*^*flox/flox*^ mice (Ctr mice) first were treated with AOM/DSS to establish colon tumors and then injected with TAM and sacrificed. Macroscopically, colorectal tumors presented in the middle and distal colon both in *Atg5-*deficient mice and Ctr mice. As shown by hematoxylin and eosin staining, while both mice similarly showed tubular adenomas or well-differentiated adenocarcinomas and signs of malignancy, such as mitotic figures and cellular and nuclear polymorphism, some tumors of the *Atg5-*deficient mice were partially hollowed out, as shown in Fig. 2b. As expected, *Atg5* exhibited negative staining throughout the majority of the colorectal tumor cell in *Atg5-*deficient mice (Fig. 2b). Immunohistochemistry showed positive staining of p62 in tumor cells both in Ctr mice and *Atg5-*deficient mice, and p62 accumulation was especially elevated in *Atg5-*deficient tumors, suggesting successful reduction of autophagic activity (Fig. 2c).

Immunoblotting showed increased expression levels of cleaved PARP, cleaved caspase 3, and BiP in colon tumor tissues of *Atg5-*deficient mice compared with Ctr mice (Fig. 2d). These results indicated that autophagic inhibition induces apoptosis in colon tumor cells. As shown in Fig. 2e, the length of the colon and body weight, which are commonly used to measure the severity of intestinal inflammation, were not significantly different between Ctr and *Atg5-*deficient mice. Although the number of colon tumors was not significantly different between Ctr and *Atg5-*deficient mice, the tumor maximum (4.5 mm in Ctr and 3.5 mm in *Atg5-*deficient mice, *p* = 0.030) and total sizes (28.1 mm in Ctr and 20.7 mm in *Atg5-*deficient mice, *p* = 0.036) were significantly smaller in *Atg5-*deficient mice than in Ctr mice.

In immunohistochemical analysis, the Ctr mice exhibited global and strong positive staining for PCNA and Ki67 in colon tumor cell nuclei. In contrast, the colon tumors of the *Atg5-*deficient mice exhibited partial negative staining for PCNA and Ki67 (Fig. 2f). Ki67-positive nuclei were decreased quantitatively in *Atg5-*deficient mice (Fig. 2g). Taken together, inhibition of autophagy in colon epithelial cells exerted an antitumor effect.

### Induced p53, caspase3, and UPR activation by autophagic inhibition in AOM/DSS-derived colon tumors

**Fig. 3:**
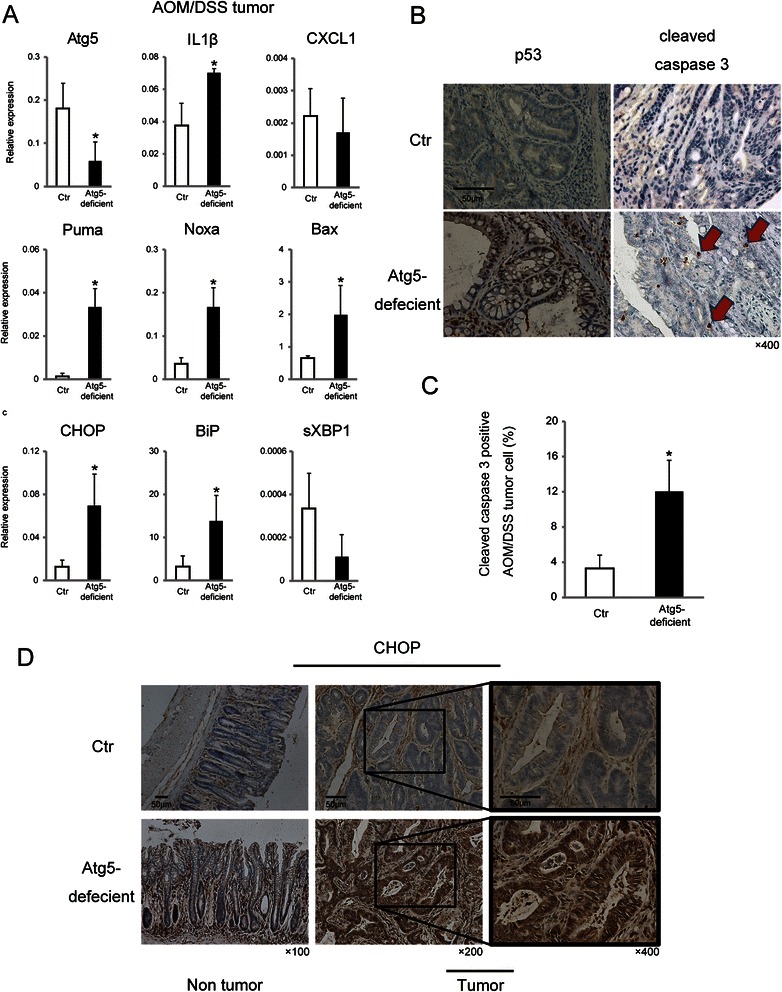
Atg5 inhibition-induced p53, caspase 3, and UPR activation in colon tumors. **a** Relative expression of the indicated mRNAs extracted from AOM/DSS-derived tumors in Ctr mice and *Atg5-*deficient mice 7 days after TAM injection. GAPDH was used as an internal control. Data shown are means ± SEM (*n* = 3). *; *p* < 0.05 by the Mann–Whitney *U*-test. **b** Representative immunohistochemical images of p53 and cleaved caspase 3 staining in AOM/DSS-derived colon tumors from Ctr mice and *Atg5-*deficient mice. Scale bar, 50 μm. Original magnification, ×400. Arrows indicate cleaved caspase 3-positive cells. **c** Cleaved caspase 3-positive tumor cells in Ctr mice and *Atg5-*deficient mice were counted per field. Data shown are means ± SEM (*n* = 3). *; *p* < 0.05 by the Mann–Whitney *U*-test. **d** Representative immunohistochemical images of CHOP in AOM/DSS-derived colon tumors and non-tumor colon mucosa in Ctr mice and *Atg5-*deficient mice. Scale bars, 50 μm. Original magnification, ×100/200/400

Since the p53 tumor suppressor inhibits cell growth and induces apoptosis, we hypothesized that p53 activation and subsequent apoptosis was the mechanism underlying the antitumor effects induced by the autophagic inhibition shown in Fig. 2. The relative expression levels of p53-related mRNAs, such as PUMA, Noxa, and Bax, and of ER stress-related mRNAs, such as CHOP and BiP, were up-regulated in the tumors of *Atg5-*deficient mice (Fig. 3a). IL1-β mRNA was also up-regulated. CXCL1 and sXBP1 mRNA expression did not differ significantly. Immunohistochemical analysis showed accumulation of nuclear p53 and cleaved caspase 3, a cell death-related factor downstream of p53 (Fig. 3b). Cleaved caspase 3-positive cells were elevated quantitatively in *Atg5-*deficient mice (Fig. 3c). The expression of CHOP was also strongly up-regulated in tumor cell nuclei of *Atg5-*deficient mice, according to immunohistochemical staining (Fig. 3d). Therefore, in colorectal tumors, the loss of autophagic activity increased p53 activation and UPR activity, followed by induction of apoptosis by cleaved caspase 3 and CHOP.

### Autophagic inhibition exerts antitumor effects in p53 wild-type cells

**Fig. 4:**
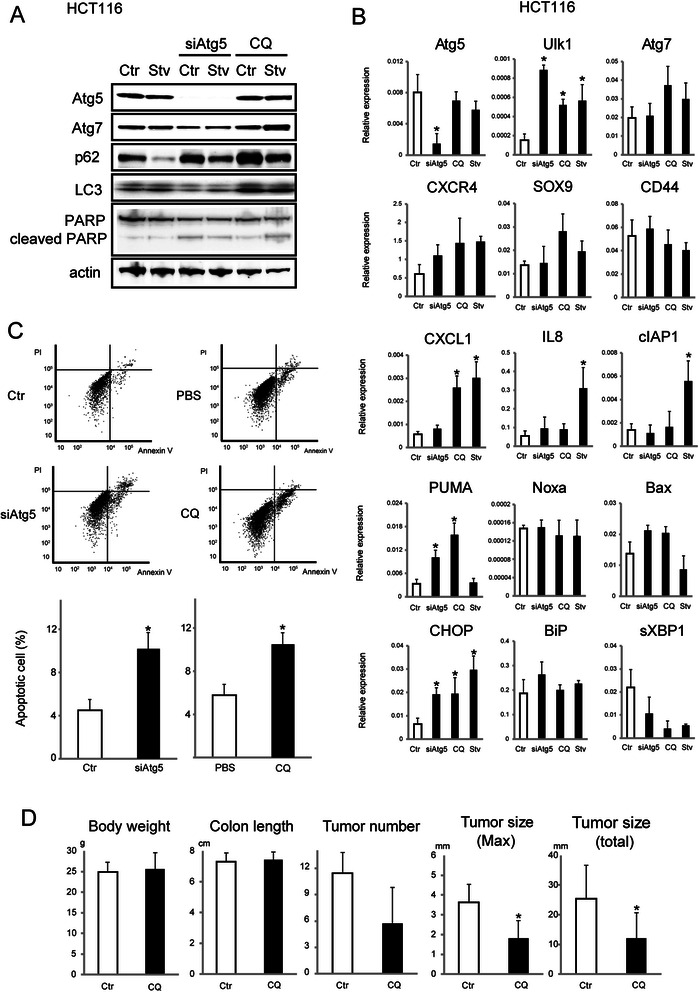
Antitumor effects exerted by autophagic inhibition in p53 wild-type colon cancer cells. **a** Immunoblot analysis of the indicated proteins in HCT116 cells transfected with non-silencing control siRNA (Ctr) or siRNA targeting Atg5 (siAtg5) for 72 h or treated with 100 μM CQ with or without amino acid starvation (HBSS medium, Stv) for 24 h. **b** Relative expression of the indicated mRNAs extracted from HCT116 cells transfected with non-silencing control siRNA (Ctr) or siRNA targeting Atg5 (siAtg5) for 72 h or treated with 100 μM CQ with or without HBSS medium (for amino acid starvation; positive control, Stv) for 24 h. Data shown are means ± SEM (*n* = 3). *; *p* < 0.05 by Dunnett’s multiple comparison test. **c** (top) Representative FACS analysis images are shown. HCT116 cells were transfected with non-silencing control siRNA (Ctr) or siRNA targeting Atg5 (siAtg5) for 72 h or treated with control PBS or 100 μM CQ for 24 h. Propidium iodide (PI)- and Annexin V-positive cells were counted using a FACS analyzer. (bottom) Apoptotic cells (Annexin V-positive cells) were counted using a FACS analyzer. Data shown are means ± SEM (*n* = 3). *; *p* < 0.05 by Welch’s *t*-test. **d** Body weight, colon length, tumor number, and tumor size were determined in AOM/DSS-treated wild type C57BL/6 J mice 7 days after three CQ treatments (*n* = 5) or control PBS treatment (*n* = 7). Data shown are means ± SEM. *; *p* < 0.05 by the Mann–Whitney *U*-test

Next, we treated the HCT116 colon cancer cell line (p53 wild type status) with CQ to examine the effects of autophagic inhibition. HCT116 cells were treated with a non-silencing control siRNA (HCT116 Ctr), siRNA targeting Atg5, or CQ in HBSS (to induce amino acid starvation conditions; positive control, Stv). Western blot analysis indicated that inhibition of autophagy increased cleaved PARP levels (Fig. 4a). We evaluated whether Atg7 reduction was induced by Atg5 inhibition, since a previous report showed that Atg7 reduction induced apoptosis [29]. Atg5 inhibition did not cause a significant reduction in Atg7, as assessed by immunoblotting (Fig. 4a).

We examined p53 and ER stress-related molecules by real-time RT-PCR (Fig. 4b). As previously shown [30], autophagic inhibition up-regulated Ulk1 (the mammalian orthologue of yeast Atg1) mRNA. While the mRNA expression levels of Noxa, Bax, BiP, and sXBP1 were not significantly different, those of PUMA and CHOP were up-regulated by autophagic inhibition.

We were also interested in the effect of autophagic inhibition on reported virulent transformation markers, such as CXCR4, SOX9, and CD44, and on chemokines, such as CXCL1 and IL8, since various reports have indicated that these markers [31–33] and inflammation [34] are associated with cancer initiation. The mRNA levels of these molecules were not significantly altered by autophagic inhibition, with the exception of the up-regulation of CXCL1 mRNA by CQ.

Subsequently, we examined apoptosis driven by autophagic reduction using FACS analysis (Fig. 4c). The number of apoptotic cells was significantly higher in autophagy-inhibited than in control HCT116 cells (10.2 % in Atg5 siRNA-treated and 4.5 % in control cells, *p* = 0.0086, and 10.7 % in CQ-treated and 6.2 % in PBS-treated cells, *p* = 0.0043).

To examine the *in vivo* effect of CQ treatment, AOM/DSS-derived colorectal tumors of wild-type B6 mice were treated with PBS, as the control (*n* = 7, male), or CQ (*n* = 5, male). As shown in Fig. 4d, the length of the colon and body weight were not significantly different between Ctr and CQ-treated mice. Although the number of colon tumors was not significantly different between Ctr and CQ-treated mice, the tumor maximum (3.7 mm for Ctr and 2.3 mm for CQ-treated tumors, *p* = 0.040) and total sizes (23.4 mm for Ctr and 7.6 mm for CQ-treated tumors, *p* = 0.036) were significantly smaller in CQ-treated mice than in Ctr mice. These results indicated that autophagic inhibition by siRNA and CQ treatments has an antitumor effect.

### Autophagic and UPR inhibition in p53 mutant cells

**Fig. 5:**
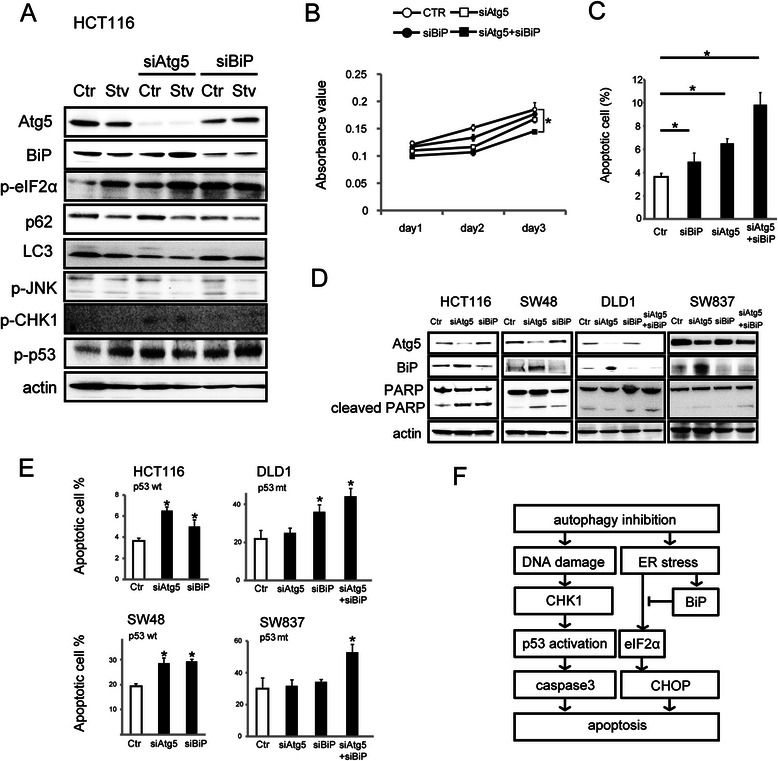
Autophagic and UPR inhibition in p53 mutant cells. **a** Immunoblot analysis of the indicated proteins in HCT116 transfected with non-silencing control siRNA (Ctr) or siRNAs targeting Atg5 (siAtg5) or BiP (siBiP) for 72 h with or without amino acid starvation (HBSS medium, Stv) for 24 h. **b** The growth curve of HCT116 cells transfected with non-silencing control siRNAs or siRNAs targeting Atg5 or BiP or Atg5 plus BiP (co-transfection), determined using a cell counting kit. **c** The number of apoptotic HCT116 cells transfected with non-silencing control siRNAs or siRNAs targeting Atg5 or BiP or Atg5 plus BiP (co-transfection), determined using a FACS analyzer. Data shown are means ± SEM (*n* = 3). *; *p* < 0.05 by Dunnett’s multiple comparison test. **d** Immunoblot analysis of Atg5, BiP, PARP, and cleaved PARP in the indicated colon cancer cell lines transfected with non-silencing control siRNAs or siRNAs targeting Atg5 or BiP or Atg5 plus BiP (co-transfection). Actin was used as an internal control. **e** The number of apoptotic cells in the indicated cell lines transfected with non-silencing control siRNAs or siRNAs targeting Atg5 or BiP or Atg5 plus BiP (co-transfection), determined using a FACS analyzer. The status of p53 in each cell line is shown. Data shown are means ± SEM (*n* = 3). *; *p* < 0.05 by Dunnett’s multiple comparison test. **f** Summary of the effect of autophagic inhibition on colon cancer. Atg5 deletion causes DNA damage and ER stress. DNA damage leads to CHK1-p53 axis activation. ER stress up-regulates BiP and eIF2α activation, and BiP inhibits eIF2α activation. Finally, apoptosis induced by caspase 3 and CHOP exerts an anti-colorectal cancer effect

Finally, we evaluated whether autophagic inhibition depends on p53 status. In addition, since suppression of the UPR pathway reportedly causes apoptosis through CHOP activation [35], we evaluated UPR pathway suppression as a potential rescue treatment. As shown in Fig. 5a, inhibition of autophagic activity induced UPR activation, including elevation of BiP levels and phosphorylation of eIF2α, especially under amino acid-free conditions. In contrast, JNK phosphorylation, an alternate UPR pathway, was not up-regulated. Increased Chk1 and p53 phosphorylation indicated that autophagic inhibition caused DNA damage and p53 activation [36]. After co-transfection of siRNAs targeting Atg5 and BiP, cell growth was reduced (Fig. 5b) and, as shown by FACS analysis, the number of apoptotic cells was increased (Fig. 5c).

We treated various colorectal cancer cell lines harboring either the wild type p53 gene (HCT116 and SW48 cells) or a mutant p53 gene (DLD1and SW837 cells) with siRNAs targeting Atg5 and BiP. Immunoblot analysis showed increased expression of BiP proteins by the Atg5-targeted siRNA in each cell line (Fig. 5d). Although siRNAs targeting either Atg5 or BiP increased PARP cleavage both in HCT116 and SW48 cells, the Atg5-targeted siRNA failed to do so in DLD1 or SW837 cells. However, co-transfection of Atg5 and BiP siRNAs increased PARP cleavage in both DLD1 and SW837 cells. As shown in Fig. 5e, although both Atg5 and BiP silencing, respectively, increased apoptosis in colon cancer cells harboring the wild type p53 gene (i.e., HCT116 and SW48 cells), Atg5 silencing alone did not show an apoptotic effect in colon cancer cells mutant for the p53 gene (i.e., DLD1 and SW837 cells). Apoptosis in DLD1 cells was increased by BiP inhibition. While SW837 cells were resistant to apoptosis induced by individual inhibition of Atg5 or BiP, apoptosis in SW837 cells was increased by co-transfection of Atg5 and BiP siRNAs. These results suggest that the anti-colon cancer effect of autophagic inhibition is influenced by p53 status, and that suppressing the UPR pathway might overcome resistance to autophagic inhibition in certain colon cancer cell types.

## Discussion

In this study, using *Atg5*^*flox/flox*^*/K19*^*CreERT*^ mice and the AOM/DSS procedure, we examined whether autophagic inhibition is effective in colon cancer treatment and showed that suppression of autophagy exerts anti-colon cancer effects *in vivo*. Our results, schematically summarized in Fig. 5f, suggested that blocking autophagy has the potential to treat colon cancer through apoptosis induced by p53 activation and ER stress. In addition, we demonstrated in colon cancer cell lines that the anti-colon cancer effect of autophagic inhibition depends on p53 status, and that UPR inhibition is a prospective alternative treatment candidate for colon cancer.

Several authors have reported that autophagic inhibition has promising anti-colon cancer effects in human colon cancer cell lines [37, 38]. The mouse model used in this experiment, exploiting Cre/loxP technology, enabled examination of not only molecule-specific but also CK19-expressing cell-specific therapies. Molecular functions, including those of autophagy related-proteins, sometimes differ according to the organ or cell type, such as somatic versus hematopoietic cells. In fact, many tissue-specific autophagy ablation models using the Cre/loxP system have been published. For example, hepatocyte-specific autophagic inhibition in Alb-Cre mice resulted in multiple liver tumors, while dendritic cell-specific autophagic inhibition in CD11c-Cre mice exhibited antigen presentation dysfunction [10, 39]. Our CK19-Cre model has the advantage of greater CK19 expression in colon tumors than in normal colonic mucosa. Indeed, we showed that autophagic inhibition had a suppressive effect on tumor size but not on tumor number. Therefore, we believe that TAM-induced autophagic inhibition is a suitable model for examining colon cancer progression in a setting in which the influence on cancer initiation has been minimized. Regarding cancer initiation, knockout mouse models displaying constitutive autophagic inhibition appear to be useful. Since genome-wide association studies have implicated that autophagy related 16-like 1 (*ATG16L1*) gene polymorphisms are associated with risk of inflammatory bowel disease [40, 41], previous researchers have established autophagy-deficiency in an intestinal epithelial cell model using Villin-Cre mice [19, 42–44]. These mice showed Paneth cell abnormalities and insufficient defense against bacteria. The existence of a cascade resulting from infection and leading to inflammation and cancer has been widely accepted [34], and it is likely that inhibition of constitutive autophagy in the intestine ultimately leads to cancer initiation. However, further investigation is required to clarify the role of autophagy in cancer initiation.

With clinical use of molecular targeting agents, the development of strategies to overcome cases of drug resistance, such as patients unresponsive to anti-EGFR agents who possess specific gene mutations, such as K-RAS mutations, is a major challenge [45, 46]. In this experiment, human colon cancer cell lines harboring mutant p53 genes showed resistance to autophagic inhibition, in accordance with a previous report [38]. Although mutation of the p53 gene is a major genetic alteration in human colon cancer [47, 48], the absence of this mutation has been reported in rodent colon tumors [22, 49]. Therefore, our *in vivo* results showing anti-tumor effects by autophagic inhibition might be due to the normal p53 gene expression in this rodent model. A combination of molecular-targeted agents seems to be a valid strategy to overcome drug resistance. We assume that anti-autophagic and anti-UPR drugs have a positive interaction, since UPR up-regulation under autophagic inhibition seems to be a compensatory pathway for colon tumor cell survival.

In fact, HCT116 cells showed an elevation in apoptosis after co-transfection with siRNAs targeting Atg5 and BiP compared with transfection of the individual siRNAs (Fig. 5c). There are three major ER stress sensors: protein kinase RNA-like ER kinase (PERK), activating transcription factor-6 (ATF6), and inositol-requiring enzyme 1 (IRE1) [50]. These sensors are in a complex that contains BiP in the unstressed state. ER stress correlation with the dissociation of BiP and activates the censors [50]. Since our analysis of autophagic inhibition showed that sXBP1 mRNA and JNK phosphorylation were not up-regulated, we believe that the ATF6-XBP1 and IRE1-XBP1-JNK pathways are not critical for UPR activation induced by loss of autophagy. In contrast, CHOP and eIF2α phosphorylation were upregulated under autophagy-suppressed conditions, indicating activation of the PERK-eIF2α-CHOP axis.

In this study, using siRNA-mediated BiP silencing, we found that BiP plays roles in ER stress and subsequent apoptosis (Fig. 5a, d), as has also been reported in previous studies [35, 51]. If the activated UPR fails to alleviate ER stress in the BiP-silenced state, pathways for apoptosis including the induction of pro-apoptotic transcriptional factor CHOP can become activated [52]. We also showed that autophagic inhibition caused ER stress, leading to up-regulation of BiP protein *in vivo* and *in vitro* (Fig. 2d and 5a). On the other hand, BiP mRNA reportedly is not always up-regulated under ER stress, since BiP expression is also controlled at the translational level [53]. This is a possible explanation for our finding that autophagic inhibition did not increase BiP mRNA *in vitro* (Fig. 4a). Under amino acid-free conditions, the effect of autophagic inhibition on increased BiP protein expression was exaggerated (Fig. 5a) and is in line with previous reports showing the importance of autophagy and UPR for cell survival, especially under starvation conditions [4, 18].

In the clinical setting, the effect of molecular-targeted therapy depends on many factors, such as genomic diversity among patients, the drug’s influence on somatic and hematopoietic cells, the drug’s interactions in cancer microenvironments, among other factors. The effect of CQ treatment may differ from that of genetic inhibition of autophagy. For example, CXCL1 mRNA expression in HCT116 cells was up-regulated by CQ treatment but not by siRNA targeting Atg5 (Fig. 5a). CQ treatment has the potential to affect hematopoietic cells directly or through stimulation of epithelial cells to induce chemokines. We also detect up-regulation of IL1-β mRNA by inhibition of autophagy *in vivo* (Fig. 3a). It is in line with the previous report that shows the up-regulation of IL1-β and IL18 in autophagy-deficient mice [54]. These cytokines might affect the immune response *in vivo* and it might be a plausible explanation of the differences between up-regulated mRNAs in the analysis of mice (Fig. 3a) and cell-based study (Fig. 4b). We believe the rodent model in this experiment could be applied to various situations using multiple mutant mouse strains and the Cre/loxP technology, as well as for *in vivo* administration of potential reagents to investigate colon cancer mechanisms.

## Conclusions

In conclusion, we showed that suppression of autophagy in colon cancer cells caused anti-tumor effects via enhanced apoptosis through p53 and UPR activation. In addition, our study implies that suppressing UPR pathway is a valid strategy when colon cancer cells with mutant p53 are resistant to autophagic inhibition.

## Abbreviations

CQ: Chloroquine
AOM: Azoxymethane
DSS: Dextran sodium sulfate
UPR: Unfolded protein response
ER: Endoplasmic reticulum
VEGF: Vascular endothelial cell growth factor
EGFR: Epidermal growth factor receptor
TAM: Tamoxifen

## References

[1] Jemal A, Siegel R, Xu J, Ward E (2010). Cancer statistics, 2010. CA Cancer J Clin.

[2] Brenner H, Kloor M, Pox CP (2014). Colorectal cancer. Lancet.

[3] Tsukada M, Ohsumi Y (1993). Isolation and characterization of autophagy-defective mutants of Saccharomyces cerevisiae. FEBS Lett.

[4] Choi AM, Ryter SW, Levine B (2013). Autophagy in human health and disease. N Engl J Med.

[5] Klionsky DJ, Abdalla FC, Abeliovich H, Abraham RT, Acevedo-Arozena A, Adeli K, Agholme L, Agnello M, Agostinis P, Aguirre-Ghiso JA (2012). Guidelines for the use and interpretation of assays for monitoring autophagy. Autophagy.

[6] Amaravadi RK, Lippincott-Schwartz J, Yin XM, Weiss WA, Takebe N, Timmer W, DiPaola RS, Lotze MT, White E (2011). Principles and current strategies for targeting autophagy for cancer treatment. Clin Cancer Res.

[7] Mathew R, Karp CM, Beaudoin B, Vuong N, Chen G, Chen HY, Bray K, Reddy A, Bhanot G, Gelinas C (2009). Autophagy suppresses tumorigenesis through elimination of p62. Cell.

[8] Su Y, Qian H, Zhang J, Wang S, Shi P, Peng X (2005). The diversity expression of p62 in digestive system cancers. Clin Immunol.

[9] Park JM, Huang S, Wu TT, Foster NR, Sinicrope FA (2013). Prognostic impact of Beclin 1, p62/sequestosome 1 and LC3 protein expression in colon carcinomas from patients receiving 5-fluorouracil as adjuvant chemotherapy. Cancer Biol Ther.

[10] Takamura A, Komatsu M, Hara T, Sakamoto A, Kishi C, Waguri S, Eishi Y, Hino O, Tanaka K, Mizushima N (2011). Autophagy-deficient mice develop multiple liver tumors. Genes Dev.

[11] Shimizu S, Takehara T, Hikita H, Kodama T, Tsunematsu H, Miyagi T, Hosui A, Ishida H, Tatsumi T, Kanto T (2012). Inhibition of autophagy potentiates the antitumor effect of the multikinase inhibitor sorafenib in hepatocellular carcinoma. Int J Cancer.

[12] Rao S, Tortola L, Perlot T, Wirnsberger G, Novatchkova M, Nitsch R, Sykacek P, Frank L, Schramek D, Komnenovic V (2014). A dual role for autophagy in a murine model of lung cancer. Nat Commun.

[13] Rosenfeldt MT, O’Prey J, Morton JP, Nixon C, MacKay G, Mrowinska A, Au A, Rai TS, Zheng L, Ridgway R (2013). p53 status determines the role of autophagy in pancreatic tumour development. Nature.

[14] Schleicher SM, Moretti L, Varki V, Lu B (2010). Progress in the unraveling of the endoplasmic reticulum stress/autophagy pathway and cancer: implications for future therapeutic approaches. Drug Resist Updat.

[15] Suh DH, Kim MK, Kim HS, Chung HH, Song YS (2012). Unfolded protein response to autophagy as a promising druggable target for anticancer therapy. Ann N Y Acad Sci.

[16] McCullough KD, Martindale JL, Klotz LO, Aw TY, Holbrook NJ (2001). Gadd153 sensitizes cells to endoplasmic reticulum stress by down-regulating Bcl2 and perturbing the cellular redox state. Mol Cell Biol.

[17] Harding HP, Novoa I, Zhang Y, Zeng H, Wek R, Schapira M, Ron D (2000). Regulated translation initiation controls stress-induced gene expression in mammalian cells. Mol Cell.

[18] Szegezdi E, Logue SE, Gorman AM, Samali A (2006). Mediators of endoplasmic reticulum stress-induced apoptosis. EMBO Rep.

[19] Adolph TE, Tomczak MF, Niederreiter L, Ko HJ, Böck J, Martinez-Naves E, Glickman JN, Tschurtschenthaler M, Hartwig J, Hosomi S (2013). Paneth cells as a site of origin for intestinal inflammation. Nature.

[20] Mizushima N, Yamamoto A, Hatano M, Kobayashi Y, Kabeya Y, Suzuki K, Tokuhisa T, Ohsumi Y, Yoshimori T (2001). Dissection of autophagosome formation using Apg5-deficient mouse embryonic stem cells. J Cell Biol.

[21] De Robertis M, Massi E, Poeta ML, Carotti S, Morini S, Cecchetelli L, Signori E, Fazio VM (2011). The AOM/DSS murine model for the study of colon carcinogenesis: From pathways to diagnosis and therapy studies. J Carcinog.

[22] Tanaka T, Kohno H, Suzuki R, Yamada Y, Sugie S, Mori H (2003). A novel inflammation-related mouse colon carcinogenesis model induced by azoxymethane and dextran sodium sulfate. Cancer Sci.

[23] Kuma A, Hatano M, Matsui M, Yamamoto A, Nakaya H, Yoshimori T, Ohsumi Y, Tokuhisa T, Mizushima N (2004). The role of autophagy during the early neonatal starvation period. Nature.

[24] Means AL, Xu Y, Zhao A, Ray KC, Gu G (2008). A CK19(CreERT) knockin mouse line allows for conditional DNA recombination in epithelial cells in multiple endodermal organs. Genesis.

[25] Hara T, Nakamura K, Matsui M, Yamamoto A, Nakahara Y, Suzuki-Migishima R, Yokoyama M, Mishima K, Saito I, Okano H (2006). Suppression of basal autophagy in neural cells causes neurodegenerative disease in mice. Nature.

[26] Ahmed D, Eide PW, Eilertsen IA, Danielsen SA, Eknæs M, Hektoen M, et al. Epigenetic and genetic features of 24 colon cancer cell lines. Oncogenesis. 2013;2. 10.1038/oncsis.2013.35 PMC3816225 24042735

[27] Baker SJ, Markowitz S, Fearon ER, Willson JK, Vogelstein B (1990). Suppression of human colorectal carcinoma cell growth by wild-type p53. Science.

[28] Moll R, Franke WW, Schiller DL, Geiger B, Krepler R (1982). The catalog of human cytokeratins: patterns of expression in normal epithelia, tumors and cultured cells. Cell.

[29] Lee IH, Kawai Y, Fergusson MM, Rovira II, Bishop AJ, Motoyama N, Cao L, Finkel T (2012). Atg7 modulates p53 activity to regulate cell cycle and survival during metabolic stress. Science (New York, NY).

[30] Yuya N, Satoko A, Kenji F, Hirofumi Y, Takeshi M, Toku K, Masaaki K, Kinya O, Yoshihide T, Shigeomi S (2009). Discovery of Atg5/Atg7-independent alternative macroautophagy. Nature.

[31] Zeelenberg IS, Ruuls-Van Stalle L, Roos E (2003). The chemokine receptor CXCR4 is required for outgrowth of colon carcinoma micrometastases. Cancer Res.

[32] Matheu A, Collado M, Wise C, Manterola L, Cekaite L, Tye AJ, Canamero M, Bujanda L, Schedl A, Cheah KS (2012). Oncogenicity of the developmental transcription factor Sox9. Cancer Res.

[33] Mulder JW, Kruyt PM, Sewnath M, Oosting J, Seldenrijk CA, Weidema WF, Offerhaus GJ, Pals ST (1994). Colorectal cancer prognosis and expression of exon-v6-containing CD44 proteins. Lancet.

[34] Karin M, Lawrence T, Nizet V (2006). Innate immunity gone awry: Linking microbial infections to chronic inflammation and cancer. Cell.

[35] Yoshida H, Haze K, Yanagi H, Yura T, Mori K (1998). Identification of the cis-acting endoplasmic reticulum stress response element responsible for transcriptional induction of mammalian glucose-regulated proteins. Involvement of basic leucine zipper transcription factors. J Biol Chem.

[36] Lord CJ, Ashworth A (2012). The DNA damage response and cancer therapy. Nature.

[37] Sasaki K, Tsuno NH, Sunami E, Tsurita G, Kawai K, Okaji Y (2010). Chloroquine potentiates the anti-cancer effect of 5-fluorouracil on colon cancer cells. BMC Cancer.

[38] Li D-DD, Sun T, Wu X-QQ, Chen S-PP, Deng R, Jiang S. et al. The inhibition of autophagy sensitises colon cancer cells with wild-type p53 but not mutant p53 to topotecan treatment. PloS one 2012;7(9):e45058. 10.1371/journal.pone.0045058 PMC3443203 23024792

[39] Lee HK, Mattei LM, Steinberg BE, Alberts P, Lee YH, Chervonsky A, Mizushima N, Grinstein S, Iwasaki A (2010). In vivo requirement for Atg5 in antigen presentation by dendritic cells. Immunity.

[40] Hampe J, Franke A, Rosenstiel P, Till A, Teuber M, Huse K, Albrecht M, Mayr G, De La Vega FM, Briggs J (2007). A genome-wide association scan of nonsynonymous SNPs identifies a susceptibility variant for Crohn disease in ATG16L1. Nat Genet.

[41] Rioux JD, Xavier RJ, Taylor KD, Silverberg MS, Goyette P, Huett A, Green T, Kuballa P, Barmada MM, Datta LW (2007). Genome-wide association study identifies new susceptibility loci for Crohn disease and implicates autophagy in disease pathogenesis. Nat Genet.

[42] Benjamin JL, Sumpter R, Levine B, Hooper LV (2013). Intestinal epithelial autophagy is essential for host defense against invasive bacteria. Cell Host Microbe.

[43] Conway KL, Kuballa P, Song J-HH, Patel KK, Castoreno AB, Yilmaz OH, et al. Atg16l1 is Required for Autophagy in Intestinal Epithelial Cells and Protection of Mice from Salmonella Infection. Gastroenterology. 2013. 10.1053/j.gastro.2013.08.035 PMC3840157 23973919

[44] Cadwell K, Liu JY, Brown SL, Miyoshi H, Loh J, Lennerz JK, Kishi C, Kc W, Carrero JA, Hunt S (2008). A key role for autophagy and the autophagy gene Atg16l1 in mouse and human intestinal Paneth cells. Nature.

[45] Douillard J-YY, Oliner KS, Siena S, Tabernero J, Burkes R, Barugel M, Humblet Y, Bodoky G, Cunningham D, Jassem J (2013). Panitumumab-FOLFOX4 treatment and RAS mutations in colorectal cancer. N Engl J Med.

[46] Amado RG, Wolf M, Peeters M, Van Cutsem E, Siena S, Freeman DJ, Juan T, Sikorski R, Suggs S, Radinsky R (2008). Wild-type KRAS is required for panitumumab efficacy in patients with metastatic colorectal cancer. J Clin Oncol.

[47] Vogelstein B, Fearon ER, Hamilton SR, Kern SE, Preisinger AC, Leppert M, Nakamura Y, White R, Smits AM, Bos JL (1988). Genetic alterations during colorectal-tumor development. N Engl J Med.

[48] Cancer Genome Atlas N (2012). Comprehensive molecular characterization of human colon and rectal cancer. Nature.

[49] Suzui M, Yoshimi N, Ushijima T, Hirose Y, Makita H, Wang A, Kawamori T, Tanaka T, Mori H, Nagao M (1995). No involvement of Ki-ras or p53 gene mutations in colitis-associated rat colon tumors induced by 1-hydroxyanthraquinone and methylazoxymethanol acetate. Mol Carcinog.

[50] Ron D, Walter P (2007). Signal integration in the endoplasmic reticulum unfolded protein response. Nat Rev Mol Cell Biol.

[51] Li J, Ni M, Lee B, Barron E, Hinton DR, Lee AS (2008). The unfolded protein response regulator GRP78/BiP is required for endoplasmic reticulum integrity and stress-induced autophagy in mammalian cells. Cell Death Differ.

[52] Sano R, Reed JC (2013). ER stress-induced cell death mechanisms. Biochim Biophys Acta.

[53] Gülow K, Bienert D, Haas IG (2002). BiP is feed-back regulated by control of protein translation efficiency. J Cell Sci.

[54] Saitoh T, Fujita N, Jang MH, Uematsu S, Yang B-G, Satoh T, Omori H, Noda T, Yamamoto N, Komatsu M (2008). Loss of the autophagy protein Atg16L1 enhances endotoxin-induced IL-1[bgr] production. Nature.

